# Measuring implementation outcomes in the context of scaling up possible serious bacterial infection guidelines: Implications for measurement and programs

**DOI:** 10.1371/journal.pone.0287345

**Published:** 2023-06-29

**Authors:** Timothy Abuya, George Odwe, Charity Ndwiga, Chantalle Okondo, Wilson Liambila, Samuel Mungai, Peter Mwaura, Kezia K’Oduol, Alice Natecho, Jesse Gitaka, Charlotte E. Warren

**Affiliations:** 1 Population Council, Nairobi, Kenya; 2 Directorate of Research and Innovation, Mount Kenya University, Thika, Kenya; 3 Kenya Paediatric Research Consortium, Nairobi, Kenya; 4 Fountain Trust Africa, Webuye, Kenya; 5 Population Council, Washington, DC, United States of America; Jhpiego, UNITED STATES

## Abstract

**Background:**

Reducing the burden of neonatal sepsis requires timely identification and initiation of suitable antibiotic treatment in primary health care (PHC) settings. Countries are encouraged to adopt simplified antibiotic regimens at the PHC level for treating sick young infants (SYI) with signs of possible serious bacterial infection (PSBI). As countries implement PSBI guidelines, more lessons on effective implementation strategies and outcome measurements are needed. We document pragmatic approaches used to design, measure and report implementation strategies and outcomes while adopting PSBI guidelines in Kenya.

**Methods:**

We designed implementation research using longitudinal mixed methods embedded in a continuous regular systematic learning and adoption of evidence in the PHC context. We synthesized formative data to co-create with stakeholders, implementation strategies to incorporate PSBI guidelines into routine service delivery for SYIs. This was followed by quarterly monitoring for learning and feedback on the effect of implementation strategies, documented lessons learned and tracked implementation outcomes. We collected endline data to measure the overall effect on service level outcomes.

**Results:**

Our findings show that characterizing implementation strategies and linking them with implementation outcomes, helps illustrate the pathway between the implementation process and outcomes. Although we have demonstrated that it is feasible to implement PSBI in PHC, effective investment in continuous capacity strengthening of providers through blended approaches, efficient use of available human resources, and improving the efficiency of service areas for managing SYIs optimizes timely identification and management of SYI. Sustained provision of commodities for management of SYI facilitates increased uptake of services. Strengthening facility-community linkages supports adherence to scheduled visits. Enhancing the caregiver’s preparedness during postnatal contacts in the community or facility will facilitate the effective completion of treatment.

**Conclusion:**

Careful design, and definition of terms related to the measurement of implementation outcomes and strategies enable ease of interpretation of findings. Using the taxonomy of implementation outcomes help frame the measurement process and provides empirical evidence in a structured way to demonstrate causal relationships between implementation strategies and outcomes. Using this approach, we have illustrated that the implementation of simplified antibiotic regimens for treating SYIs with PSBI in PHC settings is feasible in Kenya.

## Background

Approximately 2.5 million young infants die each year from preterm birth, intrapartum complications, and serious bacterial infections [1–3]. The worldwide neonatal mortality rate (NMR) is estimated at 18 per 1000 live births with sub-Saharan Africa experiencing 27 deaths per 1,000 live births [4, 5]. Although Kenya has made progress in reducing child mortality, from 41 deaths per 1000 live births in 2008 to 14 deaths per 1000 in 2014, the NMR has shown the slowest decline [6], estimated at 22 deaths per 1,000 live births in 2014 [6]. Neonatal sepsis contributes up to 20% of neonatal deaths each year [7]. Reducing this burden requires timely identification and initiation of suitable antibiotic treatment in primary health care (PHC) settings [8, 9].

Previously, World Health Organization’s (WHO) recommendation for managing infections in neonates and young infants was hospital treatment with a seven-day course of a combination of two injectable antibiotics–benzylpenicillin or ampicillin plus gentamicin [10, 11]. However, in resource-limited settings, many young infants with infection do not receive inpatient antibiotic treatment because it is not accessible, acceptable, or affordable [12–16]. Between 2008 and 2015 clinical trials demonstrated that simplified antibiotic regimens at the PHC level were effective in treating sick young infants (SYI) with signs of possible serious bacterial infection (PSBI) [9, 13, 15, 17]. This evidence contributed to WHO guidelines for SYIs with PSBI when the referral is not possible [18], with individual countries encouraged to adopt the guidelines [19]. In 2018, Kenya adopted the PSBI guidelines and incorporated it into the Integrated Management of Newborn and Childhood Illness (IMNCI) strategy.

As countries implement PSBI guidelines, there are more lessons to be learnt about effective implementation strategies and outcome measurements in low-resource settings. This necessitates pragmatic approaches such as implementation research (IR) to evaluate how countries are adopting the guidelines. IR can be defined as “a type of health policy and systems research concerned with the study of clinical and public health policies, programs, and practices, and aims to understand not only what is and is not working, but also how and why implementation is going right or wrong, and testing approaches to improve it” [20]. IR is useful in laying the foundation for successful introduction, scale up and providing an avenue for learning and adapting implementation modalities of programs. However, there are gaps in how to conceptualize and evaluate successful implementation due to lack of a clear distinction between standardized implementation, clinical and service level outcomes [21] as well as lack of clarity on how to describe implementation context and strategies [22]. These limitations pose challenges in conceptualizing and measuring implementation outcomes [21]. In this paper, we document pragmatic approaches used to design, measure IR outcomes and report implementation strategies for the “*Ponya Mtoto”* project, an IR study aimed at adapting new WHO PSBI guidelines in Kenya. Using Proctor’s work on how to conceptualize and evaluate successful implementation [21], we generated evidence on how the implementation strategies deployed influenced implementation outcomes [23]. Our paper advances the understanding of measuring implementation outcomes and strategies in the context of scaling up implementation of simplified antibiotic regimens for SYI 0–59 days with PSBI in Kenya.

## Methods

### Study context

**Fig 1:**
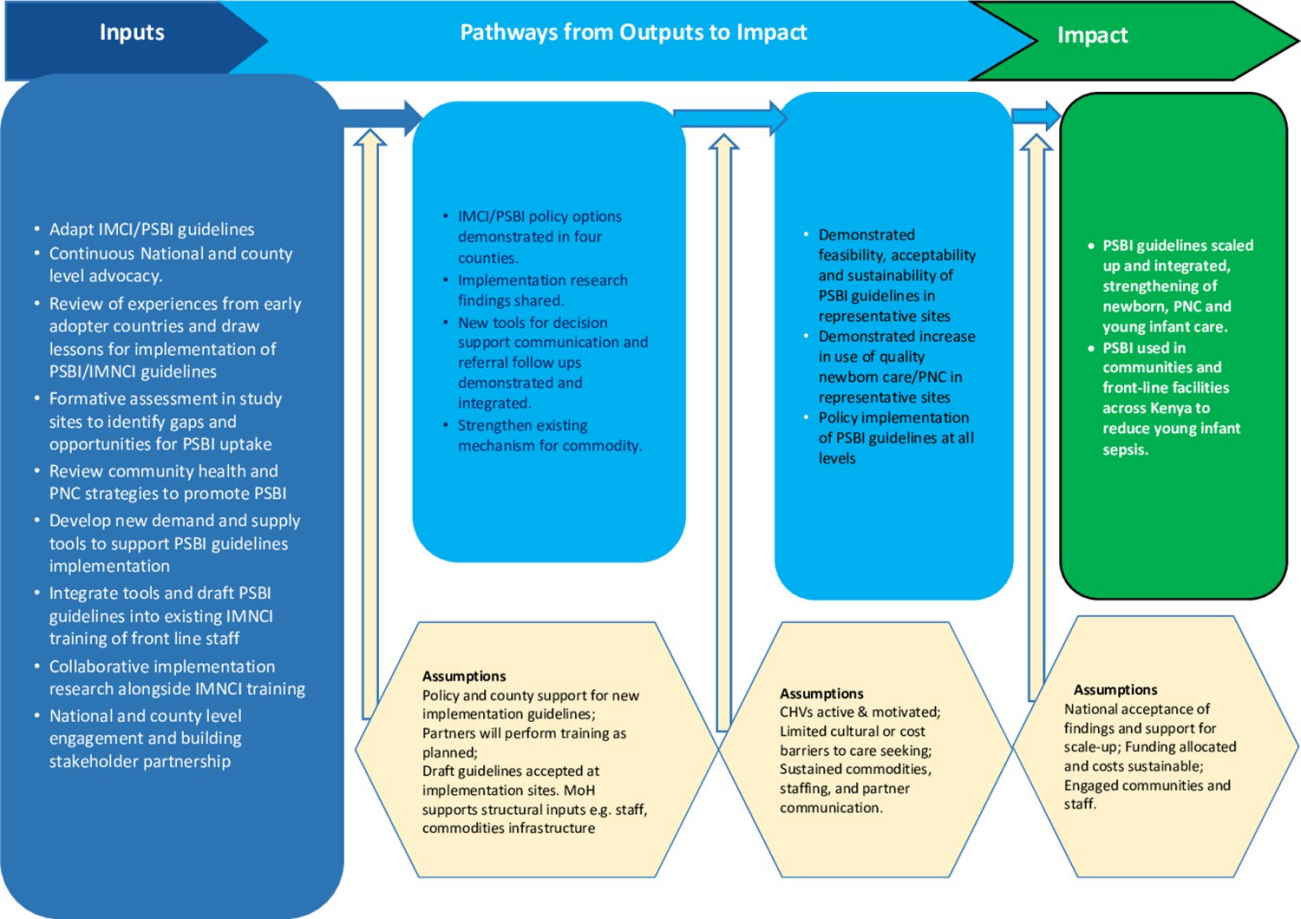
Theory of change to support adoption of PSBI guidelines in Kenya.

*Ponya Mtoto* was led by Population Council and implemented in partnership with the Kenya Paediatric Research Consortium, Mount Kenya University, and the national Ministry of Health and county health departments. The project set out to demonstrate how to adapt PSBI guidelines in PHC settings in four counties in Kenya, purposefully selected to represent diverse socio-economic and cultural settings with higher NMRs than the national average of 22 deaths/1000 live births. They include Bungoma a rural agrarian population with physical access and cultural vulnerabilities, low social economic status and with NMR of 33/1000. Kilifi, a coastal rural population representing low socio-economic vulnerability compounded with complex social and physical access challenges to health with NMR of 39/1000. Mombasa, a coastal urban setting with cultural vulnerabilities and NMR of 26/1000; and Turkana, a nomadic pastoralist lifestyle experiencing social, economic, cultural, and geographical vulnerabilities with NMR of 60/1000 live births. We developed a theory of change (TOC) (Fig 1) which enabled us design implementation strategies that supported testing assumptions throughout the project period. Proctor’s recommendation, influenced our conceptualization of measuring implementation strategies and link them with implementation and service level outcomes [23]. Measures of implementation outcomes included adoption, fidelity, and sustainability [21]. These were tested in a context of varying levels of IMNCI implementation across the four counties. In Kilifi for example, some providers had received orientation of IMNCI while in others like Bungoma, an assessment was on going on to understand the status of IMNCI to inform expanded roll out strategies. The IMNCI guidelines had already been adopted into an application that could be downloaded by providers for use. However, the capacity of providers to use them was limited by lack of adequate orientation.

Building on IR approach described by Proctor we used discrete elements of implementation strategies using seven criteria: *Actor* (who delivers the implementation strategy); *Action* (steps, process or sequences of behavior); *Action target* (where action is directed to impact); *Temporality* (order or sequence of strategy); *Dose* (intensity of implementation); *Justification* (rationale for strategy used) I*mplementation outcome affected* (defining what outcome is being affected) [23]. We linked how implementation strategies influenced implementation outcomes as precursors to service level outcome which was increased use of PSBI guidelines in PHC settings measured by tracking number of SYI identified and managed.

Data for both outcomes were generated using longitudinal mixed methods embedded in a continuous regular systematic learning and adoption of evidence within facility and county level context (Table 1). We synthesized formative data to co-create with county level stakeholders, implementation strategies that helped incorporate PSBI guidelines into routine service delivery for SYIs. This was followed by quarterly monitoring process which provided learning and feedback on the effect of implementation strategies deployed, documented lessons learnt over time and tracked implementation outcomes. Finally, we collected endline data to measure the impact on increased use of PSBI guidelines in PHC settings.

**Table 1:** Data sources and study participants over time.

Data sources	Study groups	Timing and sample
Organizational capacity assessment	Bungoma, Kilifi, Mombasa and Turkana Counties	Formative period (n– 4)
Facility Assessment	~12 facilities per county	Formative and endline Hospitals (n-10) Health centers (n-20) Dispensaries (n-20)
In-depth Interviews /case narratives	Facility based providers Female Caregivers 15–18 years Female Caregivers 19–24 years Female Caregivers Community Health Volunteers	Formative (n-14) Endline (n-13) Formative (n-11), Formative (n-12) Endline (n-7); case narrative Endline (n-7)
Focus group discussions	Female Caregivers 15–18 years Female Caregivers 19–24 years Female Caregivers 25–45 years Male Caregivers 18–34 years Male Caregivers 35 years & above Community Health Volunteers	Formative (n-7) Formative (n-12) Formative (n-6) Formative (n-3) Formative (n-4): Formative (n-7)
Process documentation	Quarterly monitoring reports Trip reports Donor reports TWG meeting notes	7 cycles (1/2019–10 /2020) implemented quarterly

*Note: Due to Covid-19 restrictions at endline fewer interviews were conducted (only those feasible by telephone). Formative phase was conducted between Aug-Sep 2018 and endline between Dec 2020-Jan 2021

### Data collection methods

Three sets of data collection took place within the project period: formative data collection (June–July 2018) was used to characterize community and facility level context, design implementation strategies to improve access to SYI services and revise our TOC (Fig 1). Iterative monitoring data (January 2019–December 2020) and endline data (December 2020–January 2021) captured infant care practices, any changes made over two years of activities while adapting PSBI guidelines, documented lessons learnt and the effect of the implementation strategies on outcomes. Data was collected by experienced research assistants with training in quantitative and qualitative data collection.

#### Facility audits, partner, and service mapping for SYI care

Facility audits in 48 facilities assessed requirements necessary to support treatment of PSBI including referral procedures for SYIs, facility infrastructure, supplies and medicines. We used a checklist to record service delivery documentation for SYIs to understand how routine data on SYI services are used for monitoring service uptake. A partner mapping exercise guided harmonization and planning of IMNCI and other SYI interventions. At endline we conducted a repeat facility assessment virtually due to Covid-19 pandemic restrictions.

#### In-depth interviews with providers and young mothers

In-depth interviews (IDI) with mothers aged 15–18 years, 19–24 years, providers, and PHC facility managers were conducted during the formative phase. Information captured included care-seeking patterns for SYI and structures to strengthen PSBI at the community level. With providers we examined facility level perceptions of care for SYIs, and challenges they face in service delivery. Endline interviews were conducted via phone with providers, community health volunteers (CHVs) and caregivers who sought care for their SYI.

#### Focus group discussions

Focus group discussions (FGDs) with CHVs and caregivers (male and female) were conducted to understand SYI care practices, care seeking patterns and decision making processes. We explored perceptions of treatment for young infants with or without referrals, the health system responsiveness to their needs and community facility linkages. No endline FGDs were conducted due to Covid-19 challenges.

### Implementing a quarterly monitoring process to track implementation

To address gaps in service delivery related to SYI as facilities adopted PSBI, we tracked progress of implementation using facility monitoring tools implemented between January 2019 to October 2020. Seven monitoring visits were conducted to document influences of service level outcomes. In each visit we captured: 1) availability of designated SYI care space; 2) availability and utilization of IMNCI/PSBI guidelines and job-aids including Newborn care handbook for PHC workers in dispensaries and health centers [24], IMNCI chart booklet, PSBI flow chart, provider pamphlet, caregiver pamphlet, PSBI assessment and follow-up forms, pediatric protocol; 3) availability of active quality improvement teams (QITs); 4) number of SYI seen at the facility; 5) number of SYI diagnosed with PSBI; 6) stock out of essential drugs (amoxicillin DT, gentamycin and Benzylpenicillin) and number of SYI with PSBI referred by CHVs.

### Ethical issues

To avoid the risk of others overhearing informants’ information, interviews were conducted in comfortable private locations, with ample time for data collection to guarantee privacy and confidentiality. Research team was trained to ensure guidance on ethical conduct was clearly understood and implemented. They were also trained to listen and observe intently without displaying any judgmental attitude. Study information was read to potential participants all above 18 years, and once they understood and accepted, participants signed the written informed consent form in the presence of a witness. There were no minors enrolled in the study. The Informed consent forms and tools were translated into Kiswahili. The research protocol was approved by the Population Council’s Institutional Review Board (PC IRB No. 838) and AMREF Ethical Review Board ESRC P430/2018 based in Kenya.

## Results

We present findings from the two-year implementation period by integrating implementation strategies recommended by Proctor [23] and link the strategies co-created during the formative phase and adapted continuously by PHC facilities to the implementation outcomes structured around adoption, fidelity, and sustainability. Table 2 illustrates key implementation strategies and outcomes which contributed to increased use of PSBI guidelines in PHC settings.

**Table 2:** Summary of implementation strategies and outcomes.

Strategies employed	Actor(s)	Actions	Target of action(s)	Temporality	Dose	Justification	Service level outcome measured	Implementation outcome
Designating areas for newborn care	Facility management	Designate a specific area or expansion of space for managing SYI	Improve efficiency of service delivery	Process began after dissemination of formative findings	Sustained over time	Co-creation process identified barrier for service delivery	Providers are treating SYI using PSBI guidelines	**Adoption**- the extent to which providers are utilizing PSBI strategy to treat SYI
Triaging of SYI	Facility management	Advocating for more providers where applicable Use CHV to support triaging Supportive facility management processes and teamwork Prioritizing treatment for SYI, introducing emergency triaging for the severely SYIs and improved assessment at all entry point where SYI are seen within the facility	Reduce burn out among providers Improve timely access to service for SYI	Quarterly review of changes made over time	Quarterly over implementation period	Complex service delivery arrangements causing delays to timely receipt of care, inadequate human resources	Eased caregiver’s access to SYI services and facilitated prompt care and helped prevent burnout Increased number of providers managing SYI and number of PSBI cases treated in PHC	
Management of medicines, and commodities for SYI	County and sub county management Study team	Redistribution of medicines to facilities with need and inclusion of key antibiotics for PSBI in counties’ forecasting, and procurement system. This included improving ordering and follow ups for the drugs like Amoxicillin DT Purchase drugs using funds from other programs such as Linda Mama Monitoring stock out trends using monitoring tools and feedback session to stimulate discussions on efficient ways of managing stock Ensuring availability of oxygen for SYI and functional resuscitation area in outpatient	Efficient management of medicines for SYI			Availability of medicines and commodities facilitate uptake of PSBI guidelines but reported stock outs for amoxicillin due to various reasons such as challenges in distribution of commodities due to lack of transportation and inconsistency in supplies of antibiotics	Reduction on average days of stock-out of essential antibiotics and increased number of PSBI cases seen	
Support facilities to functionalize quality improvement activities	Facility managers Study team	Strengthen QIT and support changes suggested by QIT Quarterly monitoring of QIT activities and changes suggested	Continuous improvement of quality of services for SYI	Quarterly review of QIT and changes made over time	Quarterly over time	Sub optimal functionality of QIT teams or some facilities having only one provider	Functionality of quality improvement system	
Emphasis on use of protocol, guidelines, and job aids	Facility managers and providers Study team	Adoption, distribution, and use of treatment protocols and job aids to create demand for SYIs services Use of flow charts, CME on PSBI, Induction by study team, encouraging providers to download and use IMCI guideline on mobile apps and emphasis on correct documentation of services provide to SYIs	Improve knowledge of providers on assessment, classification, and treatment of SYI Demystify cause of illness and reinforce caregivers understanding on treatment of SYI adherence to follow up	After development of materials and quarterly review of use of materials	Quarterly over implementation period	Knowledge gaps on assessment, classification, and treatment of SYI with PSBI Incomplete filling of assessment forms due to knowledge gaps on PSBI assessment	Provider adherence to guidelines (ability to assess, classify, and treat with recommended regimen, documenting the process) Use of community follow up forms for PSBI cases as evidenced by the number of SYIs followed up after treatment Client adherence to treatment regimen specifically return visits for day 4) Perception on why caregivers don’t come back, among those who came back, what facilitates return.	**Fidelity**-Degree to which the PSBI strategy were implemented as per guidelines.
Support in skills and confidence strengthening among health providers and CHVs as well as institutionalize skills updates for providers using blended learning approaches	Providers and local senior clinical team Study team	Continuous capacity strengthening activities using blended learning approaches mentoring, on job training, CPD and use IMNCI application Advocacy for budgetary allocation for training Strengthening Community linkages by motivating CHVs for referrals made, provision of small incentives or attaching CHVs to care givers for follow up and ensuring availability of referral forms Advocate for more providers and identify IMNCI focal persons to coordinate the process of adoption	Improve knowledge of providers on assessment, classification, and treatment of SYI	After year one of project period	Continuous process and as need arose	Gaps in knowledge on use of PSBI guidelines Trained staff going for studies, leave or new posted staff not trained in PSBI/IMNCI leading to gaps that forces facilities to use interns and trainees due to inadequate staff, contributing to workload and breaks the process of care when trained staff are absent Weak link between community and facilities	Provider adherence to guidelines (ability to assess, classify, and treat with recommended regimen, documenting the process)	
Increasing visibility and integration of PSBI in existing guidelines	Providers and managers Study team	Improving awareness at community level via community dialogues, use of mentor mothers in identification and management of SYI Integrating tracing caregivers of SYI with HIV programs who conduct defaulter tracing and Integration of PSBI services at the MCH service points Including funds for referrals in the facility budget and pre-referral counselling for mothers and caregivers Incorporation of PSBI in service charter Support the inclusion of PSBI treatment guidelines in IMNCI guidelines; newborn care handbook using global evidence	Increase visibility and integrate PSBI guidelines at all levels	During formative stage and quarterly review of process of integration of guidelines	Continuous process	Integrating PSBI into routine service structures will improve prompt treatment of SYI and overall reduction on morbidity and mortality due to bacterial infections	Improved level of awareness on SYI services including referral process from the community Number of SYI identified and referred to facilities by CHVs Improve compliance when referred for admission Integrated PSBI into existing routine service delivery structures	
Identifying challenges, generating, and tracking local solutions	Providers and managers Study team	Identifying challenges and generating solutions Strengthening community-facility linkages feedback processes including follow-up of SYIs after treatment is given Advocating for implementation of local solutions at various levels using evidence from other settings	Improve bottlenecks of service delivery points	Quarterly review of challenges, solutions implement over time	Quarterly over implementation period	Bottle neck of service delivery arrangements causing delays to timely receipt of care, inadequate linkages between community-facilities	Increased number of community referrals made to link facilities Improved documentation of community referral in the facilities and challenges and opportunities of strengthening referral and use of tools Number of changes and strategies made at facility level as part of QITs and their effect on improving care for SYI.	**Sustainability**-Extent to which the PSBI is institutionalized within the national and county health system and routine service provision.

### Strategies to improve adoption of PSBI

We defined adoption of PSBI guidelines as the extent to which providers working within a PHC facility are using PSBI guidelines to treat SYIs in the context of IMNCI whose implementation levels varies in the country. In response to the gaps and complexities of facility arrangements around management of SYIs, facilities adopted strategies to enhance uptake of PSBI guidelines. This included identifying bottlenecks and generating solutions that created opportunities for ‘doable’ steps resulting in improved efficiency of care. Four implementation strategies deployed included:

#### a). Designating places for newborn care

Formative findings illustrated that PHC facilities had limited space and did not have adequate mechanisms for identifying and managing SYIs promptly. During co-creation workshops, providers and managers confirmed this and designed several approaches to overcome this barrier. A common response was to designate a specific area for managing SYIs:

*“One is creation of more space. Initially we used to be in a smaller room with two clinicians. So, there was congestion, and the patient flow was not smooth… So, with time they gave us a bigger room, which is very spacious now. It can even accommodate two examination couches… two clerks with tables… So at least there is privacy when you are dealing with one mother, the other one cannot hear your conversation” Provider*.

Re-organizing service delivery points also supported prompt initiation of care and ability to manage SYIs in critical conditions with minimal challenges:

*“You know like in our facility, we first sat and discussed, we created a room, whereby a clinician sees only under five but before they used to queue together with the adults. At least we initiated that, and we could see in case of any emergencies in under-fives they can be taken care of fast, and even the spread of infection to the infants has been reduced” Provider*.

#### b). Triaging in support of PSBI management

During the formative phase, complex service delivery arrangements caused delays to timely receipt of care partly due to insufficient staff to support triaging. To alleviate this problem for example, 8 facilities; 4 in Bungoma and 4 in Kilifi used CHVs to assist with triaging SYI. Facility managers advocated through various county level forums for more providers, which led to increased staffing at some referral facilities and PHC facilities. Moreover, supportive facility management processes and teamwork helped prevent burnout:

“*When I came to this facility*, *we advocated getting more clinicians and so currently we have a good number whereby we can rotate freely without getting burnout while treating SYI*. *I can’t say we are badly off…but when one of us is not around… that is when we really struggle …but most of the time we are three of us… there is not much shortage of staff” Provider*.

These changes eased caregiver’s access to SYI services and facilitated prompt care as envisaged in the PSBI guidelines.

*“Okay, the support I got; firstly, the doctor acted fast. He left all his activities… in a quick manner… Besides that, I have been noticing the difference… because you find when the child goes to the triage and the temperatures are being checked… they also check for other stuff. If on that day the parent was last in line, they put her at the front… so they attended to me in a prompt manner… especially that one doctor… he is the one I mostly see handling things in a prompt manner” Case Narrative, Mother*

#### c). Management of medicines, and commodities for SYI

We supported facilities to track stock out trends over time for recommended medicines for managing SYI. The data was used during feedback sessions to stimulate discussion on efficient ways of managing commodities within the county and sub county teams. Availability of medicines and commodities facilitated uptake of PSBI guidelines enabling providers to manage SYI effectively:

*“And if you find the child has pneumonia, … when you give the Amoxicillin DT it works, because they come after maybe 48 hours and they come back… you find the child is better” Provider*.

**Fig 2:**
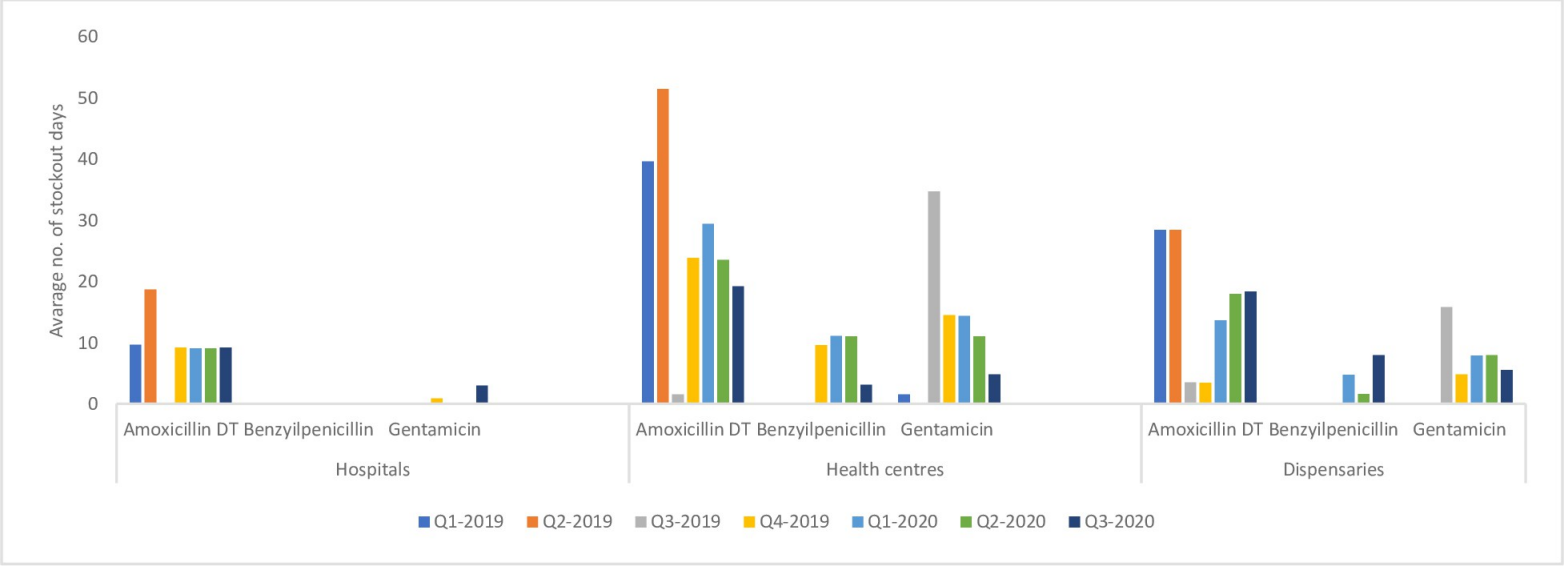
Number of stockout days for essential antibiotics- Amoxicillin DT, benzyl penicillin and gentamicin 2019–2020.

Stock-out trends illustrated as the average number of days facilities lacked essential antibiotics for management of SYI are presented in Fig 2. Overall, there was reduction on the average number of days of stock-out of essential antibiotics in study facilities across all counties. Hospitals across the four counties rarely experienced stock out of gentamicin and benzylpenicillin. However, the longest duration of stock outs of amoxicillin DT were experienced in the first and second quarter of 2019 owing to indiscriminate prescription as many providers had not been trained on its use. In PHC facilities, stock-outs for gentamycin and benzyl-penicillin were reported in quarter 4 of 2019 and throughout 2020 partly due to the outbreak of the Covid-19 pandemic where the supply chain was interrupted.

Two key approaches were used to ensure continuous supply of essential SYI medicines and commodities, including redistribution of medicines to facilities with need and inclusion of key antibiotics for PSBI in the counties’ forecasting, and procurement system. These efforts led to improved availability of antibiotics coinciding with a significant increase in the PSBI cases reported at PHC level.

#### d). Strengthening quality improvement teams

Initially *Ponya Mtoto* recognized that some facilities did not have QITs partly due to sub-optimal functionality of the teams or some facilities having only one provider. During co-creation sessions, some facility managers suggested strengthening the functionality of the QITs, subsequently increasing number of facilities with functional QITs-Table 3. Facilities with active QITs reviewed data and identified gaps in management of SYIs and recorded notable changes in adopting PSBI guidelines. Suggestions included checking medicine expiry dates through proactive forecasting and monitoring the completeness of patient records. *Ponya Mtoto* discovered that there were challenges for providers in documenting management of SYIs, due to limitations in newborn and child registers that did not include the age of the SYI. The study team subsequently worked with National Child Health Technical Working Group to advocate for and introduce registers for the newborns and young infants up to 2 months old. In addition, simplified clinical forms were introduced by the study team for providers to document management of SYI.

**Table 3:** Quality improvement activities.

Quality improvement	Baseline (n = 48)		Endline (n = 48)	
**% of facilities**	**48**	** **	**48**	** **
With a quality improvement team	40	(83.3)	42	(87.5)
**QIT with TOR**	**27**	(56.3)	**37**	(77.1)
**% of facilities that uses**	**48**		**48**	
Kenya quality model for health (KQMH)	21	(43.8)	23	(47.9)
Client-Oriented, Provider-Efficient services (COPE)	17	(35.4)	9	(18.8)
Standards-Based Management and Recognition (SBMR)	14	(29.2)	16	(33.3)
SAFECARE model	17	(35.4)	12	(25.0)
Leadership course	19	(39.6)	5	(10.4)
**Functionality of QIT (meeting in last quarter)**	48		48	
None	9	(18.8)	12	(25.0)
1–5 times	37	(77.1)	31	(64.6)
6–12 times	2	(4.2)	5	(10.4)
**% Facilities with**	**48**		**48**	
Quality improvement plan in place	31	(64.6)	33	(68.8)
Staff participating on CME for newborn care	20	(41.7)	26	(54.2)
Staff participating in QI learning last year	25	(52.1)	31	(64.6)
Documented best practices by the QIT	21	(43.8)	34	(70.8)
**% Facilities reporting**	48		48	
Support supervision of maternal health	36	(75.0)	37	(77.1)
Support supervision for newborn health	33	(68.8)	37	(77.1)
Supervision provided feedback	41	(85.4)	47	(97.9)

### Strategies to improve fidelity of PSBI

Fidelity was defined as the degree to which the treatment regimen was implemented as per PSBI guidelines. We assessed the extent in which providers adhere to the assessment, classification, as well as treatment schedule and use. From the user perspective we explored reasons for non-adherence among caregivers in return for the scheduled visit on day 4.

#### a). Emphasis on use of protocol, guidelines, and job aids

Provider knowledge gaps on assessment, classification, and treatment of SYI with PSBI identified during the formative phase indicated the need to develop materials to aid providers in service provision. The study team distributed materials in all facilities included IMNCI flow charts, client and provider job aid and pediatric clinical protocols. Job-aids for caregivers and families helped demystify the cause of illness and reinforce caregivers understanding on treatment of SYI. Initial monitoring visits to facilities showed that some providers were not using the guidelines effectively, prompting the study team to emphasize its use during subsequent quarterly monitoring visits and included reported use of guidelines in the monitoring tool, partly contributing to improvement on use of guidelines. Protocols, guidelines, and materials placed prominently, and easily accessible in-service delivery points also improved their use.

*“We have the charts placed in the consultation rooms, so every clinician when they are orientating new clinicians will use those charts to explain to them”, Provider*.

#### b). Continuous capacity strengthening activities

One key assumption identified during the development of the TOC (Fig 1) was that county teams would train health providers on IMNCI and integrate PSBI guidelines onto the existing platforms, but the formative data suggested that very few providers had been inducted. Our initial approach advocated for counties to implement updates and trainings from their budgeted annual plans or through implementing partners. However, it was evident toward the end of the first year that this was unlikely to happen due to several reasons. 1), counties had not prioritized funds for IMNCI that year, 2) partner mapping conducted during the formative phase indicated that many partners had not prioritized child health issues, 3) the period coincided with IMNCI assessments by partners to then develop IMNCI training plans.

In view of these challenges, and following discussions with county health representatives, we modified our implementation strategy and introduced other approaches to inducting providers in PSBI management during monitoring visits. We advocated use of trained county level IMNCI and senior clinical staff to support providers in continuous professional development (CPD) during structured monitoring visits. This helped providers clarify approaches to classification, and treatment and use of job aids. The monitoring teams also encouraged facilities to adopt various CPD approaches including on the-job-training or mentoring on IMNCI as need arose. This was in addition to encouraging them to use a digital IMNCI application platform developed by the ministry of health that enables self-learning on managing children using IMNCI guidelines and enrolling providers willing to be part of local community of practice (CoP) designed to reinforce learning on better management of SYIs. Continuous emphasis on the need for counties to budget for trainings was conducted during quarterly feedback sessions. Overall, providing opportunities for county teams to review challenges faced and generate solutions on capacity strengthening enabled them to incorporate changes easily. This facilitated regular CPD sessions which empowered providers to use PSBI guidelines when managing SYI and increased their confidence in symptom identification, classification, and treatment.

*“Some were trained on PSBI while some have done the on-the-job training on PSBI and IMNCI, also, on availability of drugs and provision of services. Then we have guidelines in place on prescription and how to educate mothers on the administration of drugs. We have service provider and caregiver pamphlets. We have booklets for review that help healthcare workers” Provider*.

**Fig 3:**
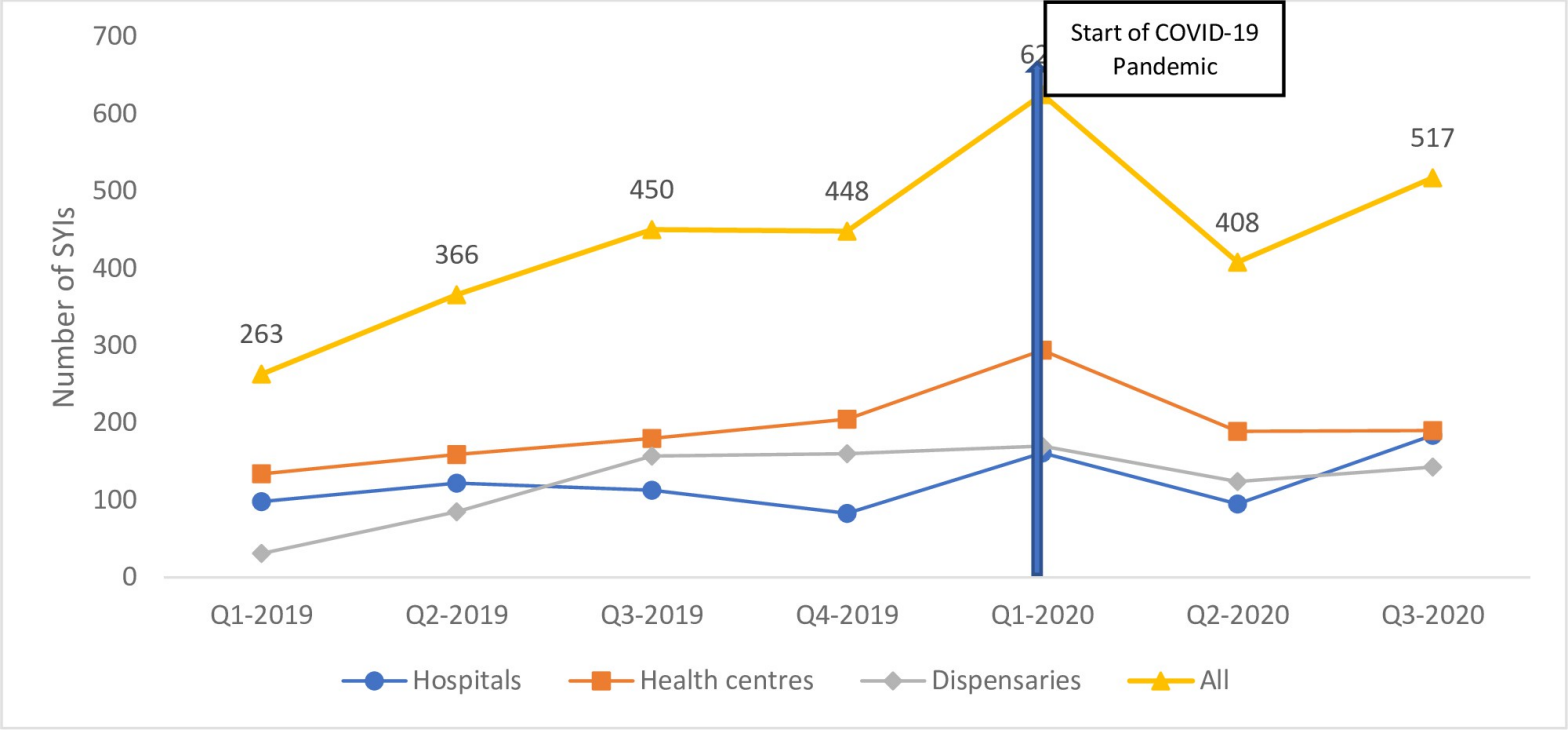
Number of SYIs with PSBI identified and treated, 2019–2020.

Resulting outcomes of these strategies were tracked using monitoring data which showed gradual improvement of identification and classification of SYIs with PSBI (Fig 3).

### Drivers of fidelity to PSBI guidelines

Formative findings indicated gaps in coordination, adequate linkages between community and PHC as well as poor documentation of SYI. We tested the assumption of the role of proper coordination between community and facilities on improving adherence to PSBI guidelines. Our findings illustrated the value of supervisory support, and improved documentation on SYI as a facilitating feature of adherence to PSBI guidelines. For example, supportive facility management processes ensured providers were given on-site technical input and their challenges were responded to during facility visits. During the quarter preceding the endline survey, nearly 80% of PHC facilities reported having been supervised and given feedback on issues observed, which provided opportunities to strengthen their capacity (Table 3).

Continuity of care through CHVs follow up was instrumental in improving linkages and follow up of SYIs. The ability of CHVs to visit SYIs at home and sensitize community members on the need for referral facilitated adherence to treatment.

*“Previously…we didn’t have CHVs… of which we have them now and they are really moving and visiting the homes, so in case of any SYI …they refer to the facility, at times they accompany … so at least there is a big change because previously those children visiting the facility… you could not even know from the nearest village that they were sick” Provider*.

**Fig 4:**
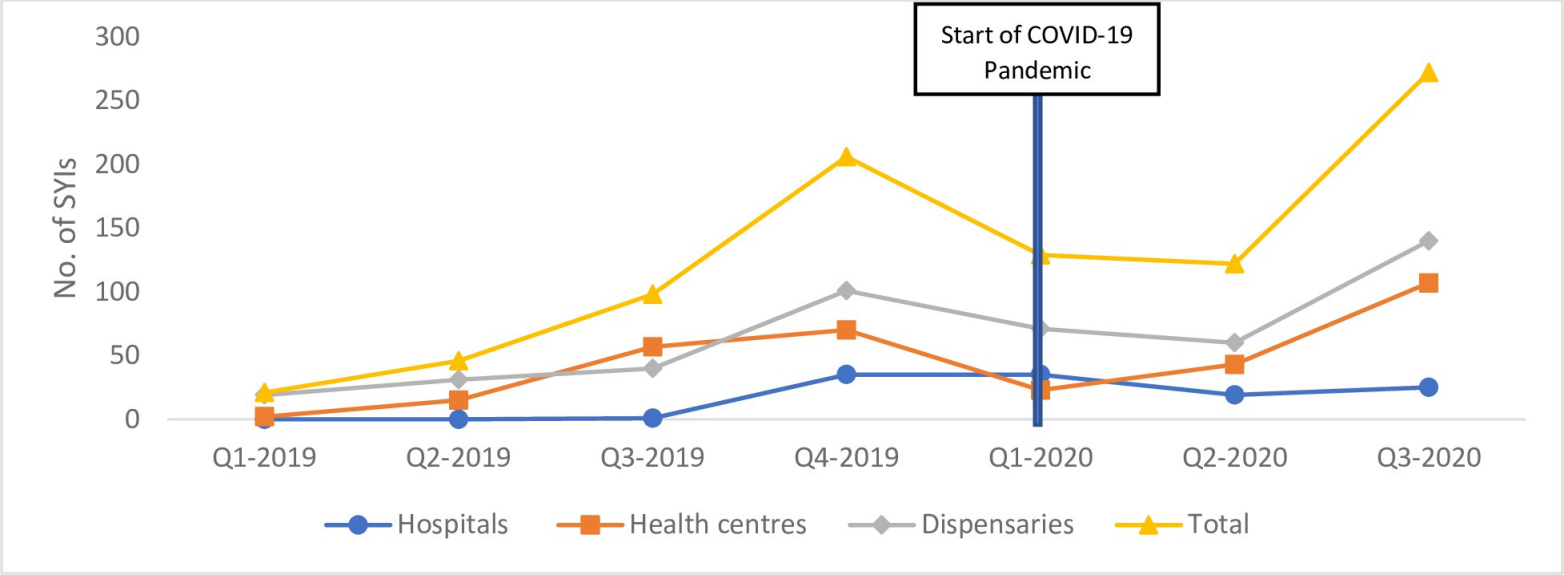
Number of PSBI cases identified by CHVs and referred to the facility, 2018–2020.

Following the introduction of the PSBI guidelines, we saw an increase in number of families seeking care for their SYIs- especially at PHC level see Figs 3 and 4. Moreover, at the caregiver level, the cost of the new PSBI regimen was considered cheaper in terms of out-of-pocket costs and allows mothers time to attend to social demands when using outpatient treatment. Although caregivers did incur costs associated with transport and medicines (if not available at facility), this was still considered favorable as opposed to referral or inpatient treatment.

*“The extent is that the percentage of mothers who have been coming for treatment as advised is big, because using the outpatient is cheaper compared to inpatient care, they have accepted because you see them coming back until the last review, and I think it allows them to take care of other responsibilities at home” Provider*

Although we observed positive changes in adherence from both the provider and client perspectives, there were several reasons for non-adherence to the treatment regimen specifically the scheduled return visit on day 4. The first set of factors related to non-adherence was logistical challenges including long distances to the facility, transport cost and/or lack of transport to the facility. In remote settings such as in Turkana, the direct transport can be as high as $80 which makes it difficult for families to bring their SYI back to complete their PSBI treatment:

*“Yes, long distance to a health facility, lack of money to pay for transport and health services in some facilities, scarcity on means of transport (few motor bikes, poor infrastructure). We use KES 2000 to and fro to the facility if using motorbike totaling to KES 8000 in all visits”, caregiver, completed visit*.

Other direct financial costs are linked to stockouts meaning caregivers are expected to buy medicines elsewhere. This together with other requirements such as buying ‘consultation materials’ such as notebooks whose price range between $3–5 cents, delay timely treatment since not everyone can afford them.

*“I remember telling you that some are unable to pay for services because whenever we have shortages with some drugs, they cannot buy another thing they come from remote areas where it requires a vehicle to reach the facility. They are pastoralists… so, it takes them days to reach here because they come on foot.” Provider*.

In other instances, families lose income on days when they seek care for their infants especially women who earn from casual labor. This in addition to other social costs that limit completion of PSBI treatment where caregivers expressed challenges of having to leave other children under someone’s care, increasing costs associated with seeking care for SYI. In situations where caregivers lack people to support other children, this delayed scheduled visits. Finally, caregivers may not complete the PSBI regimen because they see improvement in their infant’s health and don’t think it is necessary

*“….… you can come back and find she has cooked for them… so you buy her sugar, you buy her milk, and you thank her. Of course, you have no money to offer her, but you have bought her something small as a gesture of appreciation on her staying with your kids”, Caregiver*.

*“Sometimes when you ask them, they try to explain, “I was just looking for someone to come look after my children as I come to the hospital." That’s their challenge always” provider*.

“*So*, *the two injections calmed him and when I returned for the checkup on the fourth day*, *he was doing better*. *So*, *on the 8*^*th*^ *day I had been told to go back*, *but I found the baby had gotten well within the six days*, *he had no fever” Caregiver*.

### Strategies to improve sustainability

Sustainability in this context is defined as the extent to which PSBI guidelines are maintained over time or institutionalized within a service delivery setting. To explore sustainability, we examined changes made over the implementation period to improve identification and management of SYIs and ways in which PSBI management has been institutionalized as part of routine young infant care. This was one of our impact outcome (see Fig 1).

#### a). Identifying challenges, generating, and tracking local solutions

Over the course of implementation period, we observed that providers and sub county/facility managers increasingly identified challenges and addressed them with local solutions to streamline quality improvement and care for SYI. We documented changes related to structural improvement of services ranging from provision of medicines (antibiotics) commodities (oxygen, pediatric ambu bag, nebulizer machine) and equipment, (incubators, and weighing scale) to infrastructural changes such as rearranging spaces for triaging and managing SYIs. Other changes on service delivery included strengthened SYI referral system.

Consistent documentation of SYIs seen in facilities, treated and /or referred was poor initially and continued to be challenging for some health providers. There was poor use of clinical PSBI clinical form (developed with ministry of health-MoH) where forms were lacking important information such as date the client was seen; dosage given, while others had incorrect entries. Elsewhere, providers completed the registers but did not fill the clinical forms and vice versa. The study team encouraged proper use of clinical forms and worked with facility managers to ensure the laminated colored dosage charts were mounted strategically in the consultation areas to improve use. CHVs client referrals were also not documented due to shortages of referral booklets, and incomplete feedback loops. In many facilities, there was no one dedicated to handle referral forms and filing them to designated files as mothers come through different service points and in the process, the referral slips got lost. The study team focused on streamlining documentation using clinical forms as means to improve the follow-up loop. Even when the clinical forms were used, there were still follow-up gaps due to caregiver failure to return with their young infant on Day 4.

#### b). Increasing visibility and integration of PSBI in existing guidelines

At the system level there were efforts to ensure PSBI guidelines were integrated into IMNCI. This started at national level in February 2018 when the MoH embarked on the revision of the national IMNCI guidelines. MoH attendance at the global WHO meeting on PSBI in Ethiopia in January 2018 accelerated the decision to adopt the PSBI strategy and incorporate into national documents. Subsequently a series of advocacy meetings within the four counties took place during which they updated their annual work plans to integrate IMNCI/PSBI within routine activities. The WHO meeting outputs galvanized local efforts and facilitated national teamwork to continue providing guidance in revising training and service delivery materials on the use of simplified antibiotic treatment regimens of SYI at PHC level. This together with packaging evidence generated from previous research trials and the *Ponya Mtoto* formative phase in form of technical briefs, provided an avenue for sustained efforts to institutionalize PSBI guidelines.

To increase visibility of PSBI at community level, existing community dialogues sessions enabled CHVs provide information on management of SYI to increase awareness of PSBI while others utilized existing mentor mothers (peer mothers who support in HIV activities) in identification and management of SYI. Some CHVs integrated tracing caregivers of SYI with other specific programs like HIV who conduct defaulter tracing. The endline assessment shows that most CHVs continued to provide services such as health education on care for children at home for both well and sick children, follow up visits, pre-referral treatments, referrals, supporting providers in convincing those with severely SYI to accept admission, and assisting caregivers in the identification of danger signs:

*“For the small children… we always tell them that small children should not be treated at home, and if she displays any signs whatsoever… the child should be rushed to the hospital at once… so that she can get treatment … and a government hospital is the best …” CHV*

CHVs efforts resulted in a better understanding and improved awareness of SYI services including the referral process from the community. Fig 4 presents trends in the number of SYI identified and referred to facilities by CHVs. Overall, the number of SYIs referred from the community to a facility gradually increased throughout 2019 before declining in quarter 2 in 2020 then rose again in quarter 3 of 2020 partly due to the COVID-19 pandemic. Similar patterns were observed in both health centers and dispensaries, however, the number of SYI referred to hospitals by CHVs gradually increased in 2019 then plateaued in 2020 perhaps due to reduced referalls. Proper understanding and cooperation amongst caregivers, especially those who take in advice from the CHVs, created trust for effective referrals.

*“In accordance with the knowledge we have spread in our community, the mothers have accepted the importance of services provided to their children once they come to the hospital…one of them is the drugs and instructions they are given after getting services. When you go to the mother’s home, she will explain… if you ask her what kind of drugs, she has been given… she will bring it to you… and if you ask her how she was told to administer it… she will explain it to you” CHV*.

## Discussion

This study aimed to document pragmatic approaches used to design, measure and report implementation strategies and outcomes while adopting PSBI guidelines in Kenya. We used Proctor et al’s seven criteria (actor, action, action target, temporality, dose, justification, implementation outcome) to illustrate the link between implementation strategies and outcomes. Our findings show that by characterizing implementation strategies in detail and linking them with implementation outcomes, help illustrate the pathway between implementation process and outcomes [23]. Below we discuss how Proctor’s recommendation enabled us advance measurement of implementation strategies and IR and helped us draw out several lessons and insights on key elements to consider for scaling up PSBI guidelines in PHC settings.

### Lessons for measuring implementation outcomes

Although defining and measuring implementation strategies is a useful approach to advancing IR measurement, not all the seven criteria of; actor, action, targets of the action, temporality, dose, implementation outcome affected and justification of the strategy [23], may be utilizable for each IR context. We demonstrate several pragmatic challenges in using the seven criteria to highlight lessons for future IR.

First, although one must define implementation strategy elements *apriori*, when co-designing and using adaptive learning, there are changes in the strategies made over the implementation period to test various assumptions of your ToC and adjust implementation strategies to optimize results. We co-developed and continuously reviewed our ToC with national and county health officials, health facility providers, CHVs and communities, to re-evaluate assumptions, and consider new assumptions that needed to be tested. This was in the context of overcoming challenges and ensuring solutions were locally generated and appropriate for a given context. Programs that embrace co-design processes and engage local users have illustrated successful outcomes [24]. However, this poses challenges of measuring temporality and dose. For example, when designing the dose needed to generate effectiveness of implementation, variability in dose or intensity of implementation strategies limits your ability to map pathway to effective outcomes. In our case amount of time spent with providers to provide technical input during onsite visits, intensity of capacity building, and the frequency of *ad hoc* feedback from supervisors varied with different contexts making it difficult to track its influence on outcomes. Another example relates to justification of the strategy. In our case most of our strategies were derived from the formative assessment, structured site visits and monitoring necessitating adjustment of the implementation strategy over time. This is described in literature as determinants of practice [23]. The challenge is how to demonstrate their effectiveness given the different timing they are introduced in the implementation period. A few studies on PSBI have attempted to demonstrate the effect of identification of solutions, steps taken and the effect on the outcomes of interest [24–26]. Others described the solution and challenges but did not link them with the outcomes [27, 28], illustrating the complexity of linking strategies to outcome.

The challenge of dose and justification of strategy could be resolved by carefully designing a monitoring and documentation system while balancing this with the implementation process. Our efforts show that there is value in ensuring that the monitoring system does not overburden implementing teams and does not create tension with the data collected routinely through the health information system. Greater efforts should therefore be focused on aligning documentation process with the routine health information system where applicable.

The second lesson is around the complexity of defining implementation strategies which in itself can be viewed as interventions [29]. For example, capacity strengthening activities to increase fidelity of the PSBI guidelines can be considered an intervention as well as an implementation strategy, demonstrating the need to clearly define implementation strategies [23]. Previous studies reporting the feasibility of implementing PSBI guidelines in PHC settings have described interventions used but did not link them to both service level and implementation outcome[19, 24–28]. Neither did these studies report implementation strategies that generated these outcomes. Defining implementation strategies and distilling them from discrete intervention elements (even when they are multifaceted) enables one to track how strategies lead to various service level outcomes of interest.

The final lesson is around inclusion of sustainability outcomes which should be tracked at the onset and integrated over the implementation period [30]. In our case, we managed to track contextual changes made to improve uptake of PSBI from the time the country adopted the guidelines to the decision to incorporate PSBI into IMNCI guidelines. We also documented the process of enabling PSBI guidelines as routine facility or community procedures and sustain it. However, due to the short implementation period (about 18 months) we were not able to examine ‘niche saturation’—the extent to which an evidence-based intervention is integrated into all subsystems of an organization [30], limiting the understanding of sustainability outcome. Assessing niche saturation would require more time and sustained follow up often beyond short project periods.

### Key consideration for scaling up PSBI guidelines

Through the process of using the seven elements described by Proctor [23], we have demonstrated that it is feasible to implement PSBI in PHC. However, there are several considerations that will strengthen scaling up PSBI guidelines within the existing PHC structure in Kenya and integrate it within the national IMNCI program. We outline six areas to be considered when scaling up PSBI guidelines in a country context such as Kenya.

- *Effective investment in continuous capacity strengthening of providers and CHVs through blended approaches is a critical driver for improved care for SYI at PHC facilities*: These may combine mentorship, on the job training, supervisory visits, skills workshops, access to online clinical information, which will improve skills and knowledge uptake by providers and support adoption of PSBI and integrate into IMNCI program. These approaches are potentially less costly and will ensure providers continue providing services without interruption and improve provider performance [31]. This could be structured within existing support supervision processes and scheduled skills training implemented in local venues.
- *Efficient use of available human resources to detect and treat PSBI can be achieved by assigning roles to different providers to optimize timely identification and treatment*. PHC facilities are often manned by few providers, however through documentation of number of SYIs attending treatment, such data can help advocate for more staffing or negotiate for task shifting such as including using CHVs to support SYI in triaging or other roles.
- *Improving efficiency of service areas for managing SYIs improves focus and attention by providers in identifying the critically ill young infants and instituting timely treatment*. The combination of re-organizing physical space in facilities for management of SYI and upgrading infrastructure in PHCs setting in the long term will enhance the triage process of identifying SYI easily and ensure timely treatment. This will also increase chances of caregiver’s adherence to scheduled return visits on days 4 despite challenges of return visits [32], which has also been observed in other studies including Bangladesh, India, Nigeria, Ethiopia, Pakistan, Malawi [19, 25–28, 33–36].
- *Sustained provision of commodities for management of SYI facilitates increased uptake of services at PHC level as it creates caregiver trust*. Including key antibiotics for PSBI in counties’ forecasting, and procurement system is an enabler to service uptake. Availability of supplies and drugs available at the facility, increases chances for adherence by providers and caregivers and reduces cost of seeking prescriptions elsewhere facilitating greater adoption and fidelity to the guidelines.
- *CHVs are key in community linkage and timely care seeking and support adherence to scheduled visits*: frequent updates and actively involving CHVs in management of SYI at community level by empowering them through training, provision of tools of trade, appropriate supervision and incentive structure, will enhance trust between caregivers and communities, facilitate identification of SYI, referral, linkage and follow up to ensure adherence. Continuous awareness through existing community structures (community dialogues, routine CHV home visits during pregnancy and early postnatal period) will reduce socio cultural beliefs which still play a role in care seeking practices for SYI. Socio-cultural beliefs are still a barrier to timely care seeking for SYI including influence of key decision makers such as men and mother in laws during care seeking [37, 38].
- *Transport costs remain a barrier to seeking care*, Although the broader logistical issue around transport and costs associated with care seeking limits adherence among caregivers for scheduled return visits, CHVs and providers can discuss with caregivers on preparedness strategies during postnatal contacts. In remote settings, provision of motorbikes or other means of transport to CHVs may facilitate localized referral process with an arrangement that can be mutually beneficial to both CHVs and caregivers.

### Limitations

One key limitation is the inability to measure and track treatment outcomes due to logistical challenges of follow up. We designed clinical forms at facility level to document the child status on day 8 but most patients did not return on day 8 limiting this measurement. Challenges of follow up by CHV meant that the feedback loop to patient level outcome was also inhibited. Additional challenge was the prolonged COVID-19 pandemic which affected supply chain and ability to conduct in person end line assessment which resulted to conducting fewer interviews that were only feasible by telephone.

## Conclusion

Careful design, definition of terms related to measurement of implementation outcomes and strategies enables ease of interpretation of IR findings. Using the taxonomy of implementation outcomes help not only frame the research questions required to advance implementation science but also provides empirical evidence in a structured way to demonstrate causal relationships between implementation strategies and outcomes. Using this approach, we have illustrated that implementation of simplified antibiotic regimens for treating SYIs with PSBI in PHC settings is feasible in Kenya.

## List of abbreviations

CHV: Community Health Volunteer
CoP: Community of Practice
CPD: Continuous Professional Development
FGD: Focus Group Discussion
IDI: In-Depth Interview
IR: Implementation Research
IMNCI: Integrated Management of Newborn and Childhood Illness
KEPRECON: Kenya Paediatric Research Consortium
MKU: Mount Kenya University
MoH: Ministry of Health
NMR: Neonatal mortality rates
PHC: Primary Health Care
PSBI: Possible Serious Bacterial Infection
QIT: Quality Improvement Team
SYI: Sick Young Infant
ToC: Theory of Change
WHO: World Health Organization
